# Exercise and cold exposure as dual physiological stressors in MASLD: AMPK-mediated metabolic adaptation and interorgan crosstalk

**DOI:** 10.3389/fphys.2026.1865475

**Published:** 2026-07-24

**Authors:** Shijie Wang, Yuan Gao, Yang Wang, Jingfeng Wang, Yufei Liu

**Affiliations:** 1 Graduate School, Harbin Sport University, Harbin, Heilongjiang, China; 2 Laboratory Management Center, Harbin Sport University, Harbin, Heilongjiang, China

**Keywords:** AMPK, brown adipose tissue, cold exposure, exercise, MASLD, muscle-liver axis

## Abstract

Metabolic dysfunction-associated steatotic liver disease (MASLD) has become one of the most prevalent chronic liver diseases worldwide. Its disease spectrum can progress from simple hepatic steatosis to metabolic dysfunction-associated steatohepatitis (MASH), liver fibrosis, cirrhosis, and even hepatocellular carcinoma. Despite recent advancements in targeted pharmacological therapies for MASH, limitations persist regarding applicable populations and long-term benefits. Therefore, various lifestyle interventions, including dietary management and regular exercise, remain the cornerstone of MASLD management. AMP-activated protein kinase (AMPK), as an energy sensor, coordinates lipid synthesis, fatty acid oxidation, mitochondrial homeostasis, autophagy, and inflammatory responses under conditions of energy stress, thereby representing a key molecular hub connecting exercise, cold exposure, and the ameliorative effects on MASLD. Based on a narrative synthesis of mechanistic and translational evidence, this article summarizes the effects of exercise intervention, cold exposure, and their combination on AMPK-related pathways and further elucidates the potential mechanisms in terms of hepatic lipid metabolism, brown/beige adipose thermogenesis, skeletal muscle-adipose tissue-liver interorgan crosstalk, and mitochondrial quality control. Current evidence, particularly from animal and mechanistic studies, suggests that exercise and cold exposure may regulate MASLD-related metabolic abnormalities through the AMPK/ACC/SREBP1c, AMPK/SIRT1/PGC-1α and AMPK/mTOR/TFEB pathways, as well as AMPK-related myokine/hepatokine networks. The combined intervention remains an emerging strategy; preclinical data indicate potential additive effects on energy expenditure and lipid clearance, but synergistic mechanisms, optimal temperature conditions, clinical safety, and long-term efficacy require further validation.

## 1 Introduction

With the increasing prevalence of obesity, type 2 diabetes, insulin resistance, and sedentary behavior, MASLD has become a critical intersection of chronic liver disease and cardiometabolic risk. This disease is not only associated with liver cirrhosis and hepatocellular carcinoma but also closely linked to extrahepatic outcomes such as cardiovascular disease, chronic kidney disease, and declined skeletal muscle function (EASL-EASD-EASO, 2024). Formerly known as non-alcoholic fatty liver disease (NAFLD), MASLD is a chronic liver disease characterized by excessive hepatic fat accumulation (EASL-EASD-EASO, 2024). The disease is closely associated with insulin resistance (IR), obesity, inflammation, oxidative stress, and metabolic syndrome and is particularly prominent in middle-aged and elderly populations (GBD 2023 MASLD Collaborators, 2026). Although its molecular mechanisms are gradually being elucidated, pharmacological treatment options for MASLD/MASH remain limited. Recent years have seen breakthroughs in targeted pharmacotherapy for MASH. Resmetirom has received accelerated approval from the U.S. Food and Drug Administration (FDA) for patients with non-cirrhotic MASH with stage F2–F3 fibrosis; however, its use must still be combined with dietary and exercise interventions (Guirguis et al., 2025). Therefore, lifestyle intervention remains a fundamental strategy for MASLD management. Various forms of exercise, including aerobic and resistance exercise, have been widely shown to improve hepatic steatosis, insulin sensitivity, cardiometabolic function, and physical function in the context of MASLD (Stine et al., 2023; Mambrini et al., 2024). In addition, even a single bout of exercise can acutely improve insulin sensitivity and cardiometabolic function (Marjot et al., 2025). Meanwhile, animal studies have shown that various cold exposure protocols, including continuous or intermittent exposure at 4 °C and intermittent exposure at 12 °C, can activate BAT function and increase energy expenditure (Poekes et al., 2017; Yao et al., 2017; Grefhorst et al., 2018; Zhao et al., 2022). Notably, a single acute 4 °C cold exposure was also associated with a marked reduction in hepatic fat content and improvement in hepatic lipid metabolism (Grefhorst et al., 2018). These results suggest that cold exposure may exert observed metabolic effects on obesity and hepatic steatosis. Both interventions can induce energy stress and influence AMPK activity (Penugurti et al., 2024; Feng et al., 2025; Holm et al., 2025; Palomer et al., 2025). Therefore, AMPK may serve as a key molecular node integrating the effects of exercise, cold exposure, and MASLD improvement. Previous reviews have largely discussed the effects of exercise or cold exposure on metabolic diseases separately, whereas few have provided an integrated mechanistic synthesis of the shared pathways and relative strength of evidence linking exercise, cold exposure, AMPK, and MASLD. This review aims to address three questions: first, which key pathological processes in MASLD progression are regulated by AMPK; second, through which tissues and signaling axes do exercise and cold exposure influence AMPK; and third, whether exercise combined with cold exposure may produce synergistic effects and what is the current evidence base and research gap in this area. The stage-specific pathological features and corresponding AMPK-related mechanisms across the MASLD–MASH spectrum are summarized in **Table 1**.

**Table 1:** Stage-specific pathological features and AMPK-related mechanisms across the MASLD–MASH spectrum.

Disease stage	Main pathological feature	AMPK-related mechanism
Early MASLD	steatosis, DNL, insulin resistance	AMPK/ACC/SREBP1c, fatty acid oxidation (Day and James, 1998; Strable and Ntambi, 2010; Dogra et al., 2019; Lally et al., 2019; Smith et al., 2020; Filali-Mouncef et al., 2022; Yin et al., 2023; Esler and Cohen, 2024; Shokri et al., 2024; Barbhuiya et al., 2025; Li et al., 2025a; Uddandrao et al., 2025)
Progressive MASH	inflammation, oxidative stress, hepatocyte injury	AMPK/mTOR/NF-κB, AMPK/PGC-1α (He et al., 2020; Marcondes-de-Castro et al., 2023; Ren et al., 2024; Sangineto et al., 2024; Zhang et al., 2024; An et al., 2025; Che et al., 2025; Zhao et al., 2026)
Fibrotic MASLD/advanced MASH	HSC activation, ECM deposition, fibrosis	AMPK-related PLIN5/EGR1/TGF-β mechanisms (Ha et al., 2014; Li et al., 2019; Ouyang et al., 2023; EASL-EASD-EASO, 2024; Ren et al., 2024; Yin et al., 2024; An et al., 2025; Qian et al., 2025)

### 1.1 Scope of the review and evidence grading

This article was designed as a narrative mechanistic review rather than a formal systematic review. To improve transparency and consistency, the level of evidence in **Table 2** was reassessed using a modified GRADE-like narrative framework. Evidence certainty was judged according to four domains: study type, directness to MASLD/MASH populations or outcomes, consistency across studies, and whether causal/mechanistic validation was provided. Evidence was categorized as High, Moderate, Low, or Very low/preclinical. High certainty was assigned to findings supported by multiple human randomized trials, meta-analyses, or guideline-level evidence directly relevant to MASLD. Moderate certainty was assigned to human evidence with limited sample size, indirect populations, or surrogate endpoints. Low certainty was assigned to evidence derived mainly from animal studies or indirect human physiological studies. Very low/preclinical certainty was assigned to findings supported only by single or few animal/cellular studies, mechanistic inference, or unvalidated pathways. No formal PRISMA-based systematic review, risk-of-bias assessment, or meta-analysis was performed.

**Table 2:** Detailed intervention protocols, AMPK-related pathways, and narrative levels of supporting evidence in MASLD.

Intervention	Protocol (type, intensity, duration)	Tissue/System	AMPK-related pathway	Main effects in MASLD	Evidence type	Narrative evidence level
Moderate-intensity continuous training (MICT)	Treadmill/cycling, 50–70% VO_2_ max, 30–60 min/session, 3–5 sessions/week	Skeletal muscle/liver	AMPK/PGC-1α/SIRT1	↑ mitochondrial biogenesis, ↓ hepatic steatosis, ↑ insulin sensitivity	Human + animal studies	Strong (Li et al., 2021a; Bai et al., 2023; Hu et al., 2023; Stine et al., 2023; Mambrini et al., 2024; Wu et al., 2025)
Resistance training (RT)	60–80% 1RM, 8–12 repetitions, 2–3 sets, 2–3 sessions/week	Skeletal muscle	AMPK/Mtor/PGC-1α	↑ muscle mass, ↑ insulin sensitivity, ↓ adiposity	Human RCTs	Strong (Liu and Chang, 2018; Alizadeh Pahlavani et al., 2022; Stine et al., 2023; Zhu et al., 2023; Mambrini et al., 2024)
High-intensity interval training (HIIT)	85–95% HRmax intervals, 1–4 min bouts, total 15–30 min/session, 2–3 sessions/week	Skeletal muscle	AMPK/ACC/SREBP1c	↓ de novo lipogenesis, ↓ hepatic triglyceride accumulation	Human + animal studies	Strong (Bai et al., 2023; Stine et al., 2023; Mambrini et al., 2024)
Acute aerobic exercise	Single bout, moderate intensity, 30–60 min	Skeletal muscle/liver	AMPK/PGC-1α/SIRT1	↑ acute insulin sensitivity, ↑ lipid oxidation	Human studies	Moderate (Liu and Chang, 2018; Mambrini et al., 2024; Marjot et al., 2025)
Acute cold exposure	4 °C exposure, 30–120 min single session	BAT/hypothalamus	AMPK/UCP1/PGC-1α	↓ lipogenesis, ↑ fatty acid oxidation	Animal + limited human studies	Moderate (Dijk et al., 2015; Poekes et al., 2017; Yao et al., 2017; Galic et al., 2018; Grefhorst et al., 2018; Zhao et al., 2022)
Intermittent cold exposure	4–12 °C, 1–2 h/session, 3–5 sessions/week	BAT/WAT	AMPK/SREBP1c/ACC	↓ lipogenesis, ↑ fatty acid oxidation	Mainly animal studies; indirect MASLD relevance	Low certainty; preclinical (Dijk et al., 2015; Albert et al., 2016; Labbé et al., 2016; Liu et al., 2016; Galic et al., 2018)
Chronic cold adaptation	Continuous 10 °C exposure for days–weeks	BAT/skeletal muscle	AMPK/SIRT1/UCP1	↑ mitochondrial adaptation, ↑ thermogenic capacity	Animal models	Low certainty; preclinical (Dijk et al., 2015; Albert et al., 2016; Labbé et al., 2016; Xu et al., 2019a; Castro et al., 2021)
Combined exercise + cold exposure (acute)	7 °C environment + 60 min moderate-intensity exercise	Skeletal muscle	AMPK/PGC-1α/p38 MAPK	↑ PGC-1α expression, ↑ fatty acid oxidation	Single human physiological study; non-MASLD population; surrogate molecular endpoints	Low certainty; indirect human evidence (Slivka et al., 2013; Shute et al., 2018)
Combined training protocol	Treadmill exercise + cold exposure (4–7 °C), 30–60 min/session	Muscle–fat–liver axis	AMPK/PGC-1α/irisin	↑ WAT browning, ↓ visceral fat accumulation	Animal studies only; limited replication	Very low certainty; preclinical (Jiang et al., 2022; Weng et al., 2023; Geng et al., 2025)
BAT thermogenic activation	Acute 4 °C exposure	BAT	AMPK/UCP1	↑ non-shivering thermogenesis, ↓ lipid burden	Animal studies	Low certainty; preclinical (Dijk et al., 2015; Albert et al., 2016; Labbé et al., 2016; Liu et al., 2016; Wei et al., 2017; Galic et al., 2018; Xu et al., 2019a; Castro et al., 2021)
Skeletal muscle cold adaptation	Chronic 4–6 °C exposure	Skeletal muscle	AMPK/PGC-1α/GLUT4	↑ glucose uptake, ↑ insulin sensitivity	Animal + limited human studies	Moderate (Oliveira et al., 2004; Lee et al., 2014; Blondin et al., 2015; Periasamy et al., 2017; Sepa-Kishi et al., 2017; Ruixia et al., 2021; van Gerwen et al., 2023; Zhang et al., 2025a)

Evidence certainty was reassessed using a modified GRADE-like narrative framework based on study type, directness to MASLD/MASH outcomes, consistency across studies, and causal or mechanistic validation. “High” certainty indicates support from multiple human randomized trials, meta-analyses, or guideline-level evidence directly relevant to MASLD. “Moderate” certainty indicates human evidence with limited sample size, indirect populations, or surrogate endpoints. “Low” certainty indicates evidence derived mainly from animal studies or indirect human physiological studies. “Very low/preclinical” certainty indicates evidence supported only by single or few animal/cellular studies, mechanistic inference, or pathways not directly validated in MASLD/MASH populations. This grading is intended for narrative evidence mapping and does not represent a formal GRADE assessment.

## 2 Overview of AMPK regulation in MASLD

### 2.1 AMPK regulates DNL and promotes fatty acid oxidation

*De novo* lipogenesis (DNL) has been identified as a key process driving the progression of MASLD. This process occurs mainly in hepatocytes and is triggered by excessive intake of glucose or fructose, ultimately converting surplus carbohydrates into fatty acids and triglycerides (TG) (Esler and Cohen, 2024). Although DNL is a normal metabolic pathway required for maintaining physiological homeostasis, abnormally increased DNL activity may lead to hepatic steatosis (Smith et al., 2020). Therefore, inhibition of excessive DNL activity has become an important therapeutic target for MASLD. The key transcription factors involved in DNL, sterol regulatory element-binding protein 1c (SREBP1c) and carbohydrate-responsive element-binding protein (ChREBP), promote the expression of DNL-related lipogenic enzymes (Filali-Mouncef et al., 2022; Barbhuiya et al., 2025). These two factors cooperatively regulate normal metabolic function; however, when their levels are abnormally elevated, specific cellular mechanisms are activated to terminate signal activation (Strable and Ntambi, 2010). AMPK regulates DNL through multiple mechanisms. On the one hand, it phosphorylates and inactivates acetyl-CoA carboxylase (ACC), thereby inhibiting fatty acid biosynthesis (Li et al., 2025a). On the other hand, it suppresses transcriptional regulators such as SREBP1c and ChREBP, thereby reducing hepatic lipid accumulation (Lally et al., 2019; Li et al., 2025a). In various disease models, AMPK consistently exerts regulatory effects on SREBP1c and ChREBP levels, thereby modulating DNL (Dogra et al., 2019; Yin et al., 2023; Shokri et al., 2024). These studies demonstrate the role of AMPK in targeting fatty acid synthesis. Under different physiological conditions, AMPK is indirectly regulated by silent information regulator 1 (SIRT1) through SIRT1-mediated deacetylation of liver kinase B1 (LKB1), an upstream kinase of AMPK (Tang, 2016; Anggreini et al., 2023). Studies have shown that SIRT1 knockdown in the human hepatoma cell line HepG2 leads to upregulation of ChREBP expression (Lu et al., 2024). Reports in mice have also indicated that SIRT1 suppresses SREBP1c activity and reduces hepatic lipogenesis (Chyau et al., 2020). These findings suggest that SIRT1 plays an important role in AMPK-mediated regulation of DNL. Notably, AMPK may also participate in MASLD regulation through the autophagy–lysosomal pathway. Transcription factor EB (TFEB), a key regulator of the autophagy–lysosomal pathway, controls the expression of genes involved in autophagy and lysosomal biogenesis. TFEB activation synchronously regulates both the upstream initiation and downstream degradation stages of autophagic flux, thereby restoring autophagic flux and promoting intracellular clearance (Chen et al., 2025b). The AMPK/SIRT1 pathway is an important mechanism for enhancing TFEB function. AMPK phosphorylation activates SIRT1, which subsequently activates TFEB, ultimately promoting the expression of autophagy- and lysosome-related genes and improving MASLD (Zhang et al., 2025b). Taken together, these findings suggest that the AMPK/SIRT1 pathway participates in both fatty acid synthesis and cellular autophagy regulation, mainly through modulation of the activity of factors such as SREBP1c, ChREBP, and TFEB.

### 2.2 The impact of AMPK on the “two-hit” hypothesis

The traditional “two-hit” hypothesis describes insulin resistance (IR)-driven hepatic lipid accumulation as a key manifestation of early MASLD, followed by inflammation- and oxidative stress-related injury that is closely associated with the progression to MASH (Che et al., 2025). In the early stage of MASLD, AMPK may attenuate the first-hit process by inhibiting mTOR/SREBP-1c-mediated lipogenesis and reducing hepatic triglyceride accumulation (Uddandrao et al., 2025). Evidence from high-fat diet models further indicates that activation of the IRS-1/AMPK/mTOR/SREBP-1c axis is associated with reduced lipogenic signaling and improved metabolic injury (Day and James, 1998; Uddandrao et al., 2025). Regarding the second hit, oxidative stress is a major contributor to MASLD-related liver injury, and mitochondrial respiratory activity represents an important source of reactive oxygen species (Sangineto et al., 2024). This process is regulated in part by the AMPK/mTOR/PGC-1α pathway (Sangineto et al., 2024). In the liver, excessive mTORC1 activation can promote lipogenesis and suppress autophagy, thereby contributing to lipid accumulation and hepatocyte injury (He et al., 2020). It may also aggravate chronic hepatic inflammatory responses through NF-κB-related cytokine production (Zhang et al., 2024). In monocytes from patients with MASH, increased mTOR levels have been associated with reduced AMPK phosphorylation (Marcondes-de-Castro et al., 2023). When AMPK is activated, it can suppress mTORC1 by phosphorylating TSC1/2 or inhibiting Raptor, thereby reducing lipid synthesis and hepatic inflammatory responses (He et al., 2020; Marcondes-de-Castro et al., 2023; Ren et al., 2024; Zhang et al., 2024). These findings suggest that AMPK may counteract both hits and that changes in mTOR signaling are an important factor in this process, with excessive mTOR activity contributing to MASLD progression.

The AMPK/mTOR pathway can also be activated by irisin, a key regulator of mitophagy. In a high-fat diet mouse model, irisin ameliorated hepatic steatosis by upregulating silent information regulator 3 (SIRT3) and phosphorylated AMPK, inhibiting mTOR activity, promoting TFEB nuclear translocation, enhancing cathepsin B expression, and increasing autophagic degradation capacity (Zhao et al., 2026). Notably, nutritional status differentially regulates autophagy. Under nutrient-rich conditions, mTORC1 is activated to promote anabolism and inhibits autophagy initiation by phosphorylating Unc-51-like kinase 1 (ULK1) and autophagy-related protein 13, thereby interfering with the AMPK–ULK1 interaction (An et al., 2025). Under nutrient-deprived conditions, AMPK inhibits mTORC1 activity by phosphorylating the mTORC1 regulatory components Raptor and TSC2, subsequently activating the ULK1 complex to promote autophagy. This process leads to the accumulation of autophagy receptor proteins such as p62/sequestosome 1 (p62), thereby regulating MASLD progression (An et al., 2025). Therefore, under altered nutritional or energy states, modulation of the AMPK/mTOR pathway can effectively regulate hepatic lipid synthesis, autophagy initiation, and hepatic inflammatory responses.

### 2.3 The impact of AMPK on liver fibrosis

Liver fibrosis is a late manifestation of MASLD and typically reflects the progression from simple steatosis to MASH or advanced MASLD. Perilipin 5 (PLIN5) may influence the transition from hepatic steatosis to the fibrotic stage (EASL-EASD-EASO, 2024). Elevated levels of PLIN5 can enhance glucose tolerance, induce healthy remodeling of white adipose tissue, and protect against high-fat diet–induced hepatic steatosis. Conversely, PLIN5 knockdown may lead to hepatic steatosis, insulin resistance (IR), and increased lipotoxicity, thereby accelerating the progression of MASLD to MASH (Ren et al., 2024). In MASLD models, high-fat diet–fed PLIN5 knockout mice typically exhibit exacerbated hepatic lipotoxicity, liver inflammation, and more pronounced hepatic fibrosis, although this condition may be accompanied by alterations in lipid droplet morphology (Zhao et al., 2026). Interestingly, PLIN5 knockdown also reduces AMPK phosphorylation levels (Ouyang et al., 2023), suggesting that AMPK expression is influenced by PLIN5 and that PLIN5 functions as an upstream regulator of AMPK. Notably, the role of PLIN5 appears to be inconsistent across different hepatic cell types. In the regulation of hepatic stellate cell (HSC) activation to influence liver fibrosis, PLIN5 overexpression promotes HSC activation, resulting in decreased mitochondrial ATP levels, suppressed cell proliferation, and markedly increased apoptosis (Yin et al., 2024). Conversely, PLIN5 knockdown enhances AMPK phosphorylation in activated HSCs, promoting proliferation while reducing apoptosis (Yin et al., 2024). Therefore, PLIN5 may regulate AMPK-related signaling in a cell type- and context-dependent manner, particularly during lipid droplet–mitochondria interactions and HSC activation.

**Figure 1:**
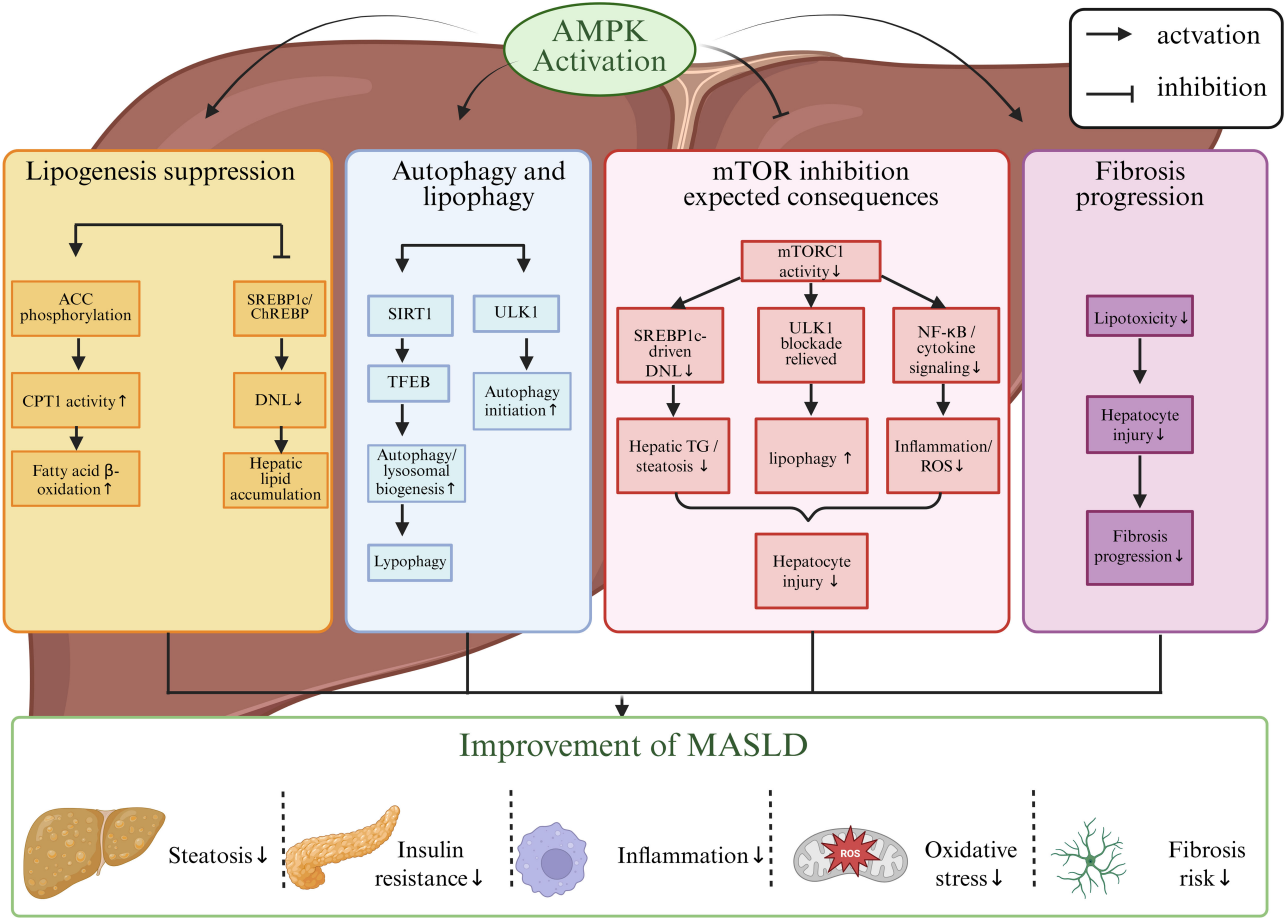
mechanistic insights into ampk-mediated regulation of MASLD pathogenesis.

Furthermore, under energy-deprived conditions, AMPK activation induces the expression of early growth response protein 1 (EGR1) in hepatocytes. In MASLD models combined with chronic obstructive pulmonary disease, EGR1 expression triggers TGF-β–driven pathways that promote liver fibrosis (Qian et al., 2025). Studies in the human acute monocytic leukemia cell line THP1 have shown that the antihypertensive drug valsartan inhibits EGR1 expression by activating the LKB1/AMPK signaling pathway, thereby stabilizing or even reversing liver fibrosis (Ha et al., 2014). Notably, under stress or specific conditions, EGR1 activation can exert protective effects in MASLD, with mechanisms related to its roles in hepatic metabolism, insulin resistance, regulation of liver inflammation, and hepatoprotection (Li et al., 2019). In summary, AMPK plays a central role in MASLD progression through lipid droplet–mitochondria interactions mediated by its downstream effectors. Both reduced PLIN5 expression and elevated EGR1 expression may accelerate hepatic fibrosis, whereas AMPK activation and its regulation of PLIN5 and EGR1 constitute key mechanisms counteracting fibrosis progression. The major AMPK-mediated mechanisms involved in lipid metabolism, autophagy, inflammation, oxidative stress, and fibrosis during MASLD progression are summarized in **Figure 1**.

## 3 Regulatory role of AMPK in MASLD during cold exposure

### 3.1 Mechanisms of cold exposure-mediated regulation of lipogenesis

**Figure 2:**
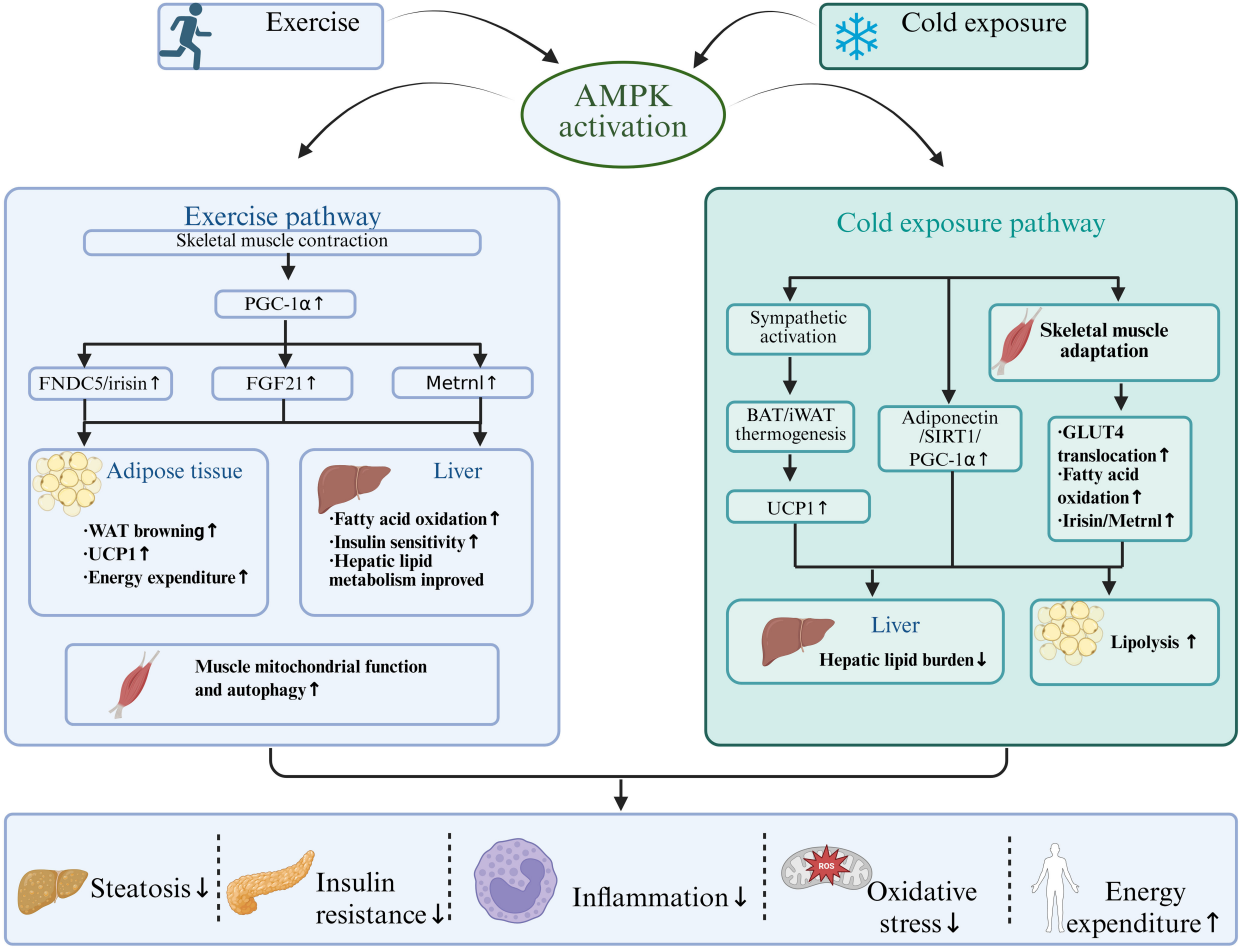
Exercise and cold exposure converge on AMPK to improve MASLD.

As illustrated in **Figure 2**, exercise and cold exposure converge on AMPK through tissue-specific pathways, thereby contributing to improvements in multiple MASLD-related metabolic outcomes. Cold exposure may regulate AMPK/mTOR expression in a tissue-specific manner. Before interpreting cold exposure studies, it is important to distinguish cold exposure from thermoneutral conditions. The thermoneutral zone refers to the ambient temperature range at which basal metabolic rate is minimal and additional thermoregulatory energy expenditure is not required; this range is approximately 30–33 °C in mice, 27–29 °C in rats, and around 20–22 °C in lightly clothed humans (Romanovsky et al., 2002; Ganeshan and Chawla, 2017; Fischer et al., 2018; Škop et al., 2020). Evidence from non-mammalian models, including chicks, broilers, and fish, suggests that chronic mild cold exposure may, under certain physiological conditions, activate AMPK in tissues such as the heart and liver, inhibit mTOR activity, downregulate lipogenic gene expression, and ultimately improve lipid accumulation and insulin resistance (IR) (Nguyen et al., 2015; Deng et al., 2020; Zhang et al., 2025d). However, these models differ substantially from mammalian thermogenic responses under cold exposure. In mice, continuous cold stimulation at 4 °C for seven days has been shown to activate mTORC1 signaling in BAT and iWAT. Meanwhile, rapamycin administration or adipocyte-specific Raptor deletion attenuated UCP1 expression and WAT browning (Liu et al., 2016). Similarly, acute cold exposure at 10 °C for 6 h activated mTORC1 in mouse BAT through the sympathetic nervous system, whereas adipose tissue-specific Raptor deletion blocked cold-induced BAT recruitment, reduced mitochondrial biogenesis, and severely impaired BAT oxidative metabolism (Labbé et al., 2016). These findings suggest that mTORC1 plays a critical role in BAT recruitment and metabolic adaptation during cold exposure. Further evidence indicates that after cold acclimation at 10 °C for 14 days, overall mTORC2 activity in BAT and iWAT may be suppressed, but residual mTORC2 activity remains important for maintaining UCP1 content and thermogenic capacity. At the same time, cold exposure can upregulate BAT mTORC1 activity and BAT/iWAT glucose uptake independently of mTORC2 (Albert et al., 2016). These findings further support the important role of mTOR signaling in thermogenesis, WAT browning, oxidative metabolism, and mitochondrial biogenesis during cold exposure. Therefore, in the context of cold-induced BAT/iWAT thermogenesis in mammals, mTOR signaling should be regarded as an adaptive thermogenic regulator rather than merely as a downstream target inhibited by AMPK. However, direct evidence remains limited regarding whether cold exposure improves MASLD in mammalian liver through the hepatic AMPK/mTOR/SREBP1c pathway.

In addition, in mice exposed acutely to 4 °C, AMPK/ACC signaling appears to be required for cold-induced appetite regulation rather than for adipose thermogenesis. Galic et al. showed that mice with non-phosphorylatable ACC maintained energy expenditure and thermogenesis under sub-thermoneutral conditions, but failed to increase food intake appropriately during fasting or cold exposure (Galic et al., 2018). Therefore, in the acute cold-exposure setting, adipose thermogenesis may be activated largely through ACC-independent mechanisms, most likely involving sympathetic–β-adrenergic stimulation, lipolysis, UCP1 activation, and mitochondrial fuel oxidation. By contrast, ACC may still contribute to cold-related metabolic adaptation by regulating fatty acid synthesis, malonyl-CoA availability, CPT1-mediated fatty acid oxidation, and chronic adipose tissue remodeling. Dijk et al. reported similar findings, showing that continuous cold exposure at 4 °C may significantly downregulate angiopoietin-like protein 4 (ANGPTL4) in brown adipose tissue (BAT) through AMPK activation, thereby enhancing lipoprotein lipase (LPL) activity and the uptake of plasma triglyceride-derived fatty acids (Dijk et al., 2015). These findings support the regulatory effects of cold exposure on lipid metabolism. In other words, different cold exposure protocols, including continuous cold stress at 4 °C and 10 °C, can activate AMPK in the hypothalamus, heart, and adipose tissues, thereby enhancing lipid oxidation and thermogenesis and improving whole-body energy expenditure. Although the roles of hepatic and skeletal muscle AMPK in lipid metabolism have been well established, direct evidence showing that cold exposure enhances AMPK activity in these tissues remains limited and requires further investigation. Therefore, in mammals, cold exposure may reduce lipid synthesis, attenuate cellular apoptosis, alleviate endoplasmic reticulum stress, and promote autophagy through the activation of AMPK- and mTOR-related pathways, suggesting its potential as a therapeutic strategy for MASLD.

### 3.2 Cold exposure-mediated thermogenic effects in BAT/iWAT

The most direct metabolic effect of cold exposure is the activation of the sympathetic nervous system and BAT thermogenesis. Under acute and chronic cold stress, both brown adipose tissue (BAT) and beige adipose tissue can consume fatty acids and glucose through uncoupling protein 1 (UCP1)-mediated non-shivering thermogenesis, promoting the transport of plasma triglyceride-derived fatty acids to thermogenic tissues and thereby reducing the hepatic lipid burden (Xu et al., 2019b; Cohen and Kajimura, 2021; Zhang et al., 2025c). AMPK participates in this thermogenic adaptation by promoting fatty acid oxidation, regulating the adiponectin/SIRT1/PGC-1α pathway, and influencing UCP1 expression. Adiponectin is essential for maintaining body temperature stability in cold environments. In adiponectin-knockdown mice exposed acutely to 4 °C cold, AMPK activity in BAT was reduced, and SIRT1 expression was decreased (Wei et al., 2017). This suggests that, under cold exposure, adiponectin may regulate BAT thermogenic activity and insulin signaling through the AMPK/SIRT1 pathway, thereby influencing lipid metabolism. In cold-acclimated Brandt’s voles, transient receptor potential (TRP) channels may participate in BAT thermoregulation through the Ca²^+^/calmodulin-dependent protein kinase II (CaMKII)/AMPK/SIRT1/UCP1 pathway (Lv et al., 2023). These findings indicate that the AMPK/SIRT1/UCP1 pathway is a key mechanism by which the body coordinates mitochondrial biogenesis and energy expenditure to regulate thermogenesis. During cold stress, PGC-1α, a downstream target of AMPK, participates in adaptive thermogenesis. Under acute 4 °C cold exposure, increased levels of PGC-1α and UCP1 in BAT promote thermogenesis (Zhang et al., 2025c). The underlying mechanism may involve cold-induced phosphorylation of PGC-1α, which increases UCP1 expression and mitochondrial biogenesis (Zhang et al., 2025c). Multiple studies have shown that upregulation of PGC-1α increases UCP1 expression in the inner mitochondrial membrane, thereby mediating UCP1-dependent thermogenesis and enhancing BAT transcriptional activity (Hou et al., 2018; Asghari Alashti and Goliaei, 2025). This suggests that PGC-1α, as an upstream regulator of UCP1, plays an important role in cold-induced thermogenesis. Notably, mTOR exerts an inhibitory effect on UCP1. Studies in chronically cold-exposed mice have shown that adipocyte mTORC2 deficiency impairs total UCP1 content and thermogenic capacity in both the BAT and inguinal white adipose tissue (iWAT) of cold-adapted mice through distinct mechanisms (Castro et al., 2021). AMPK activation appears to reverse this inhibitory effect (Xu et al., 2019a). Therefore, under cold exposure, AMPK-mediated pathways involving UCP1 and PGC-1α mainly act as regulators of chronic thermogenic potential in BAT, thereby indirectly modulating hepatic lipid metabolism.

### 3.3 Cold exposure-mediated metabolic adaptation in skeletal muscle

Skeletal muscle is also an important responsive organ for cold adaptation (Periasamy et al., 2017). Cold exposure can increase skeletal muscle glucose uptake and fatty acid oxidation through shivering thermogenesis and non-shivering metabolic adaptation (Blondin et al., 2015). In the skeletal muscle of cold-acclimated rats, AMPK activation promotes GLUT4 translocation, improves insulin sensitivity, and synergistically regulates mitochondrial biogenesis with PGC-1α (Sepa-Kishi et al., 2017; van Gerwen et al., 2023). Given that the PGC-1α/FNDC5/irisin axis is associated with skeletal muscle metabolism and sarcopenia, cold exposure–induced AMPK/PGC-1α activation may contribute to skeletal muscle cold adaptation by regulating irisin secretion (Zhang et al., 2025a). In animal models, 4 °C cold exposure significantly upregulates AMPK phosphorylation and PGC-1α expression in skeletal muscle, promotes GLUT4 expression and skeletal muscle glucose uptake, increases whole-body energy expenditure, and thereby helps prevent obesity and enhance glucose tolerance (Oliveira et al., 2004). In humans, cold exposure can induce an increase in serum FGF21 levels and enhance irisin secretion (Lee et al., 2014). AMPK and irisin can participate in adipose tissue lipolysis and the regulation of lipolytic gene expression by upregulating the activity of key lipolytic enzymes, such as adipose triglyceride lipase (ATGL) and hormone-sensitive lipase (HSL) (Gao et al., 2016; Kim et al., 2016). Since PGC-1α is an upstream regulator of the FNDC5/irisin axis in skeletal muscle (Boström et al., 2012), it is plausible that cold exposure enhances irisin-related signaling through AMPK/PGC-1α activation. However, research on the relationship between this pathway and MASLD outcomes remains limited. Long-term cold exposure at 4–6 °C in rats has been reported to increase p-AMPK, PGC-1α, and UCP1 expression and to regulate skeletal muscle cell proliferation; this effect was suppressed by the AMPK inhibitor Compound C (Ruixia et al., 2021). These findings support a role for the AMPK/PGC-1α pathway in skeletal muscle adaptation under cold conditions. Moreover, meteorin-like protein (Metrnl) secretion has been reported to increase after ice-water swimming in adults (Mu et al., 2023). Metrnl overexpression can activate AMPK-related pathways in diabetes and sarcopenia models, with effects involving autophagy, mitochondrial homeostasis, thermogenesis, lipid accumulation, insulin resistance, and inflammation (Jung et al., 2018; Lu et al., 2023; Chen et al., 2025a; Iglesias, 2025). Therefore, AMPK activation under cold exposure is crucial for skeletal muscle metabolic adaptation, suggesting that the effects of AMPK on MASLD in cold environments may derive not only from adipose tissue thermogenesis but also from enhanced skeletal muscle metabolic capacity.

## 4 Regulatory role of AMPK in MASLD via the muscle-liver axis under exercise intervention

### 4.1 Extrahepatic crosstalk mechanisms of exercise-mediated skeletal muscle in regulating MASLD

Skeletal muscle is the primary responsive tissue to exercise interventions and also an important organ for extrahepatic regulation of MASLD. On one hand, alterations in multiple myokine levels have been observed in sarcopenia and MASLD, including decreased irisin and increased FGF21 levels (Jung et al., 2021; Marjot et al., 2025). Medium- to long-term endurance exercise in both humans and mouse models can upregulate PGC-1α expression in skeletal muscle and promote the release of myokines such as irisin, FGF21, and Metrnl (Boström et al., 2012). Exercise-induced irisin release provides a mechanistic link between skeletal muscle contraction and MASLD-related metabolic regulation. Irisin, as a myokine involved in interorgan communication, may contribute to MASLD improvement by promoting the browning of white adipose tissue (WAT), reducing fatty acid influx to the liver, and enhancing energy expenditure (Scheel et al., 2022). The role of FGF21 is mainly reflected in the improvement of hepatic inflammatory responses, insulin resistance (IR), and hepatic lipid metabolism (Milani et al., 2024). Metrnl primarily improves fatty acid oxidation in skeletal muscle, lipid-induced inflammation, and insulin resistance (IR) through AMPK/PPARδ-related pathways (Jung et al., 2018). Together, irisin, FGF21, and Metrnl constitute an exercise-induced myokine network that ameliorates MASLD-related metabolic abnormalities. On the other hand, in the context of MASLD complicated by sarcopenia, reduced skeletal muscle mass, impaired mitochondrial function, and elevated inflammatory cytokines may in turn exacerbate insulin resistance and increase the risk of liver fibrosis (Marjot et al., 2025). Interestingly, reduced AMPK activation has been observed in both MASLD and sarcopenia (Zeng et al., 2020; Yang et al., 2024). In addition, PGC-1α levels associated with the size of type IIX and IIB muscle fibers are also decreased in both conditions (Kerr et al., 2024; Yang et al., 2024). In animal models, various forms of exercise, including endurance and resistance training, can induce activation of AMPK and its downstream targets—including SIRT1, PGC-1α, Nrf2, and mTOR—promoting improvements in mitochondrial function, energy metabolism, and autophagy, thereby enhancing skeletal muscle mass and functional recovery and significantly ameliorating sarcopenia (Liu and Chang, 2018; Zeng et al., 2020; Alizadeh Pahlavani et al., 2022; Zhu et al., 2023).

### 4.2 Exercise-mediated browning of white adipose tissue and tissue-selective mechanisms of action

Exercise-induced physiological adaptations are commonly accompanied by upregulation of PGC-1α expression, which in turn promotes the expression of multiple myokine-related gene products, including fibronectin type III domain-containing protein 5 (FNDC5) (De Sousa, 2024). The FNDC5 gene encodes a type I membrane protein that is proteolytically cleaved to release irisin, a hormone secreted into the circulation (Wang et al., 2022). Irisin acts on multiple tissues and organs, including adipose tissue, skeletal muscle, the liver, and the ovary, and is involved in the browning process of white adipose tissue (WAT) (Wang et al., 2022; Guo et al., 2023; Grzeszczuk et al., 2024; Khan and Wahab, 2024). Studies have shown that irisin can stimulate the expression of uncoupling protein 1 (UCP1) in white adipocyte precursors both *in vitro* and *in vivo*, promoting their differentiation into thermogenic cells (Grzeszczuk et al., 2024). This process is mediated through activation of the p38 mitogen-activated protein kinase (p38 MAPK) pathway, which is essential for WAT browning, and activation of p38 MAPK is in turn critical for PGC-1α expression (Boström et al., 2012; Grzeszczuk et al., 2024; Zhou et al., 2025). These findings suggest that the exercise-induced irisin/UCP1 pathway is an important mechanism driving adipose tissue browning. Notably, not all adipocytes undergo transdifferentiation. Experimental models have shown that adipocytes derived from visceral adipose tissue (VAT) do not respond to irisin stimulation via p38 MAPK activation, indicating that visceral adipocytes do not undergo significant browning upon stimulation of this pathway (Li et al., 2021b). However, irisin can still reduce the production of pro-inflammatory cytokines in both visceral and subcutaneous adipose tissues through other mechanisms that are not yet fully understood, thereby alleviating obesity-related chronic low-grade inflammation (Li et al., 2021b). Another important role of irisin in exercise is improving insulin sensitivity by enhancing insulin receptor responsiveness in skeletal muscle, thereby promoting hepatic glucose and lipid metabolism. Further studies have revealed that, in mouse models, the beneficial effects of regular aerobic exercise on insulin resistance and dysregulated glucose–lipid metabolism are partly mediated through the irisin/AMPK signaling pathway, which suppresses mitochondrial fission (Sánchez et al., 2022). AMPK also plays a regulatory role in irisin expression. Studies on icariin have shown that the AMPK antagonist compound C or AMPK gene silencing inhibits the effect of icariin on FNDC5 protein expression, indicating that icariin enhances FNDC5 expression via the AMPK pathway (Chen et al., 2019). Therefore, changes in AMPK activity play an important regulatory role in processes such as oxidative stress, steatosis, and inflammation in liver and adipose tissues. Overall, exercise induces irisin secretion through the AMPK/PGC-1α pathway, promoting WAT browning, increasing energy expenditure, and improving insulin sensitivity. However, irisin does not appear to induce transdifferentiation in visceral tissue; its role in the viscera is primarily associated with alleviating chronic inflammation.

### 4.3 Remote regulatory mechanisms of exercise-mediated hepatic lipid metabolism

Fibroblast growth factor 21 (FGF21) is a liver-derived endocrine hormone and a direct target of peroxisome proliferator-activated receptor α (PPARα) (Montagner et al., 2016). Under physiological conditions, hepatic basal FGF21 levels are relatively low; however, under stress conditions such as cold exposure and exercise, both hepatic and circulating FGF21 levels are significantly increased (Feng et al., 2023). Exercise also stimulates FGF21 production in skeletal muscle, which can regulate hepatic lipophagy through endocrine signaling via the circulation (Feng et al., 2023). In MASLD rats, aerobic exercise has been shown to regulate lipid metabolism through activation of the AMPK/ACC signaling pathway (Bai et al., 2023). The underlying mechanism is primarily related to FGF21, which reduces lipid accumulation and ameliorates cellular senescence by enhancing autophagy (Xu et al., 2025). Endocrine FGF21 signaling activates the AMPK pathway through two mechanisms: one involves direct signaling via fibroblast growth factor receptor 1 (FGFR1)/β-Klotho; the other involves indirect activation of AMPK in target tissues through stimulation of adiponectin and glucocorticoid secretion (Salminen et al., 2017). Studies have shown that a specific AMPK inhibitor can completely block the effects of FGF21, confirming that FGF21 induces autophagy and lipophagy in an AMPK-dependent manner (Kong et al., 2022). Subsequently, AMPK regulates downstream lipid metabolism processes by activating its downstream target SIRT1, thereby alleviating MASLD. Multiple studies have demonstrated that aerobic exercise-induced activation of the AMPK/SIRT1 signaling pathway reduces lipid accumulation, increases energy expenditure, suppresses *de novo* lipogenesis (DNL), upregulates fatty acid metabolism, and attenuates chronic metabolic inflammation in the liver (Li et al., 2021a; Hu et al., 2023; Wu et al., 2025). This pathway may also serve as a potential therapeutic target for reducing oxidative stress and improving oxidative damage (Wu et al., 2025). Recent reports have indicated that exercise-induced activation of the AMPK/SIRT1/PGC-1α signaling pathway can effectively improve hepatic glucose metabolism and insulin sensitivity (Zhang et al., 2020). In contrast, multiple *in vivo* and *in vitro* studies in MASLD models have reported dysregulation of the AMPK/SIRT1 signaling axis (Lee et al., 2025). Therefore, exercise induces skeletal muscle secretion of FGF21, which acts on the liver via the circulation and activates autophagy and SIRT1 signaling in an AMPK-dependent manner, thereby enabling remote regulation of hepatic lipid clearance. In addition, the regulatory effects of FGF21 on MASLD are also associated with mTOR signaling. Animal studies have shown that skeletal muscle-derived FGF21 enhances insulin signaling and hepatic glycogen synthesis by inhibiting mTORC1 (Yano et al., 2022). Consistent with normal physiological conditions and cold exposure, moderate-intensity exercise also regulates lipid metabolism through activation of the AMPK/ACC pathway. Specifically, exercise promotes phosphorylation of AMPK and inhibits ACC, thereby enhancing carnitine palmitoyltransferase 1 (CPT1) activity, increasing hepatic fatty acid β-oxidation efficiency, improving lipid metabolic disorders, and ultimately preventing hepatic lipid accumulation (Fang et al., 2022; Portincasa et al., 2024). Similarly, long-term aerobic exercise enhances AMPK phosphorylation, downregulates SREBP1 expression, suppresses lipogenesis, and improves lipid accumulation in the context of MASLD (Dogra et al., 2019; Bai et al., 2023; Yin et al., 2023; Shokri et al., 2024). Therefore, under exercise intervention, hepatic lipid metabolism is also regulated by AMPK-related downstream factors that modulate the expression of lipogenic enzymes and fatty acid β-oxidation processes.

## 5 Role of AMPK in MASLD under the combined context of exercise and cold exposure

**Figure 3:**
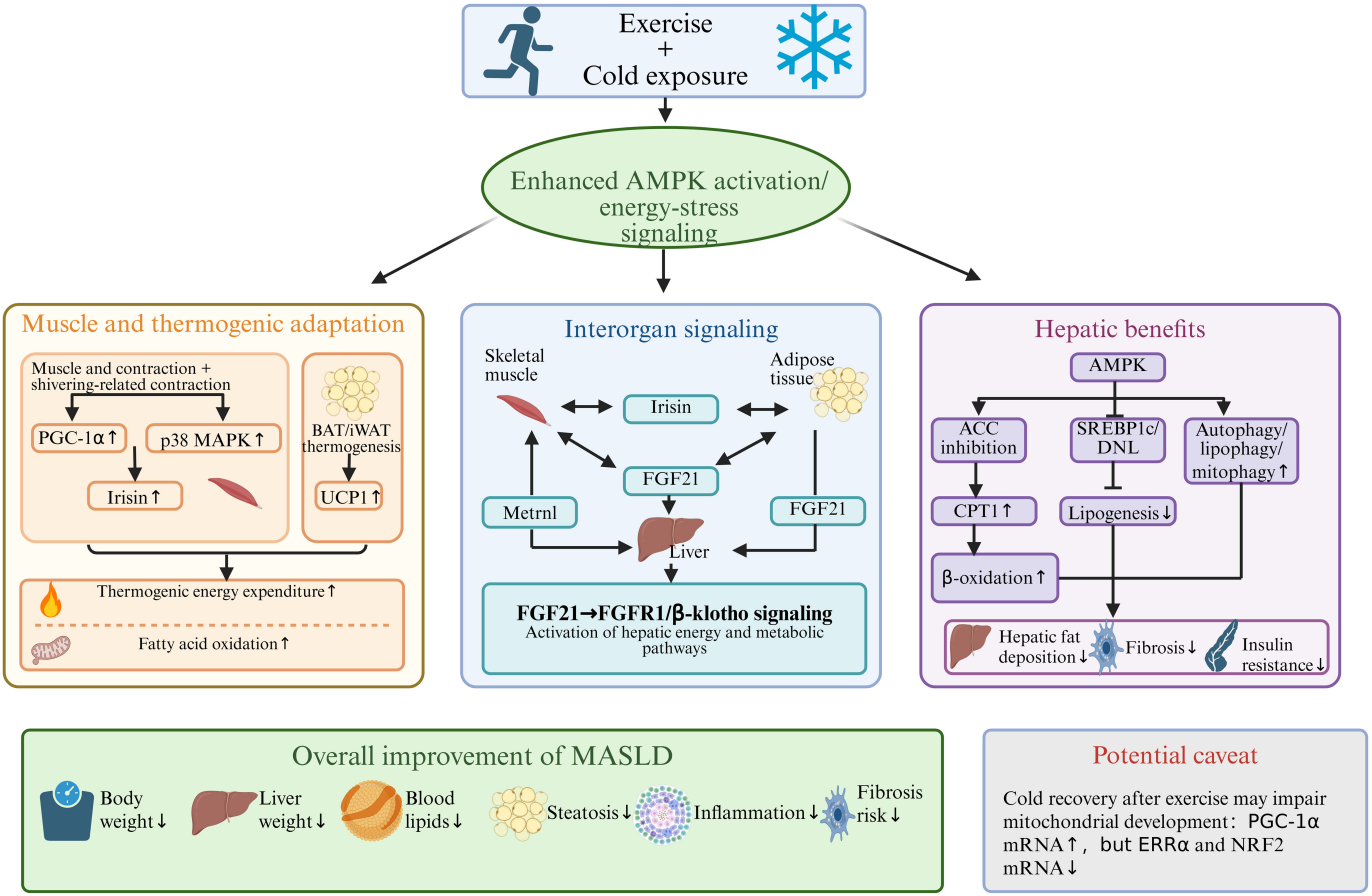
Role of AMPK in MASLD under the combined context of exercise and cold exposure.

Studies have shown that, under a 7 °C cold exposure environment, a single bout of moderate-intensity aerobic exercise significantly increases PGC-1α mRNA levels 3 hours after intervention in recreationally trained men, whereas cold exposure alone does not produce the same effect (Shute et al., 2018). Further studies have demonstrated that combined treadmill exercise and cold exposure upregulates PGC-1α and p38 MAPK protein expression, enhances skeletal muscle uptake and oxidation of serum free fatty acids, and effectively reduces subcutaneous and visceral fat mass in rats (Weng et al., 2023). Increased PGC-1α levels stimulate irisin secretion, promote upregulation of UCP1 in adipose tissue, and regulate thermogenesis via the PPAR-α/UCP1 pathway (Scheel et al., 2022). As described above, during exercise, there is a reciprocal activation relationship between AMPK and irisin, and AMPK acts as an upstream regulator of PGC-1α. We therefore hypothesize that exercise-induced improvements in lipid metabolism under cold conditions may be partly mediated by the AMPK/PGC-1α/irisin signaling pathway. These findings support the notion that combined interventions may amplify lipid oxidation and thermogenic adaptations through AMPK and its associated energy metabolic network. However, the current evidence remains insufficient to define this response as an established mechanism in MASLD. The beneficial effects of combined cold exposure and exercise are also related to shivering thermogenesis in skeletal muscle. Studies indicate that exercise-induced irisin secretion under cold conditions is derived from shivering-related skeletal muscle contractions and acts synergistically with FGF21 to enhance brown adipose tissue thermogenesis (Lee et al., 2014; Jiang et al., 2022). In one murine MASLD study, combined exercise and cold exposure reduced hepatic lipid deposition and fibrosis and was associated with increased hepatic FGFR1 and β-Klotho protein expression (Geng et al., 2025). These findings suggest that the FGF21–β-Klotho/FGFR1 axis may be involved in the metabolic response to combined intervention. Interestingly, post-exercise recovery in a cold environment may impair mitochondrial biogenesis. Studies have shown that after 60 minutes of cycling exercise, recovery in a 7 °C cold environment increases PGC-1α mRNA expression in healthy adult males, while simultaneously reducing the mRNA levels of estrogen-related receptor α (ERRα) and nuclear factor erythroid 2-related factor 2 (NRF2), which may contribute to mitochondrial dysfunction (Slivka et al., 2013). In summary, the current certainty of evidence for combined exercise and cold exposure in MASLD remains low to very low. Limited preclinical studies and acute human physiological evidence suggest that combined intervention may modulate hepatic fatty acid oxidation and DNL through AMPK-related pathways, promote mitochondrial adaptation in skeletal muscle and adipose tissue, and regulate inter-organ signaling molecules such as irisin, FGF21, and Metrnl, thereby potentially reducing the risk of lipotoxicity and fibrosis progression. However, acute human studies currently provide only indirect physiological evidence, whereas long-term combined-intervention studies remain largely limited to animal models. Therefore, the proposed effects on hepatic fatty acid oxidation, DNL suppression, lipophagy, mitophagy, and FGF21–β-Klotho/FGFR1 signaling should be interpreted as preclinical or hypothesis-generating mechanisms rather than established therapeutic pathways. The proposed AMPK-related mechanisms underlying the combined effects of exercise and cold exposure on MASLD are summarized in **Figure 3**.

However, it should be noted that sex as a biological variable should also be considered when discussing the effects of cold exposure and exercise on MASLD. Previous reports have shown that men of reproductive age have a significantly higher risk of MASLD than women, whereas postmenopausal women have an increased risk of MASLD and advanced fibrosis, suggesting that estrogen-related signaling pathways may exert protective effects against MASLD (Ballestri et al., 2017; Lonardo et al., 2019). Sex differences are also important factors influencing cold-induced thermogenesis. Human PET-CT studies have reported a higher prevalence of detectable brown adipose tissue in women than in men, and cold-induced thermogenesis appears to be stronger in premenopausal women, partly in association with estradiol levels (Cypess et al., 2009). In rodents, females generally exhibit greater brown adipose tissue mass, higher UCP1 expression, more favorable mitochondrial characteristics, and earlier activation of thermogenic responses during cold exposure (Kaikaew et al., 2021; Fernández-Peña et al., 2023). These differences may influence the degree of brown adipose tissue/inguinal white adipose tissue activation, lipid substrate utilization, and AMPK-related metabolic adaptation during cold exposure. In addition, exercise-induced skeletal muscle substrate utilization and AMPK signaling may differ by sex, depending on muscle fiber composition, training status, and hormonal status (Roepstorff et al., 2006). Therefore, current evidence derived mainly from male animals or mixed-sex human cohorts may not fully explain whether significant sex differences exist in the effects of exercise, cold exposure, and their combined intervention on MASLD. Future studies should perform stratified analyses according to sex, menopausal status, and hormonal levels, and should clarify whether combined exercise and cold exposure induce comparable AMPK activation, adipose thermogenesis, myokine/hepatokine responses, and MASLD improvement in males and females.

## 6 Clinical translational feasibility and safety considerations of exercise and cold exposure interventions in MASLD

Although experimental studies have shown that exercise and cold exposure can improve hepatic lipid metabolism and delay fibrosis progression through multiple AMPK-related pathways, thereby ameliorating MASLD, their safety and feasibility in clinical applications for MASLD should be carefully considered. Relevant studies have indicated that cold exposure increases cardiovascular burden, leading to elevated blood pressure, increased heart rate, and peripheral vasoconstriction (Ikäheimo, 2018). Exercise increases myocardial oxygen consumption and cardiac workload (Duncker and Bache, 2008). The combination of both further increases myocardial oxygen consumption and may induce adverse effects such as early myocardial ischemia (Li et al., 2025b). This highlights the importance of the intervention modality. The comparative risk profiles, cardiovascular effects, and clinical recommendations for these interventions are summarized in **Table 3**. A study by Stine et al. found that incorporating resistance training alongside either >150 minutes per week of moderate-intensity exercise or 75 minutes per week of vigorous-intensity exercise can effectively improve hepatic steatosis in patients with MASLD (Stine et al., 2023). Notably, exercise prescriptions should be individualized based on physical fitness, stage of liver disease, and comorbid conditions (Stine et al., 2023). According to ACSM risk stratification and MASLD guidelines, exercise, cold exposure, and combined interventions are more suitable for patients with early or moderate MASLD, who have no severe cardiovascular disease, decompensated liver function, or serious diabetic complications (Stine et al., 2023). Individuals with hypertension, coronary artery disease, arrhythmias, severe obesity, diabetic neuropathy, or limited mobility should undergo medical screening before intervention and avoid unsupervised, high-risk intervention modalities (Stine et al., 2023). Intervention protocols should be appropriately adjusted according to individual differences. Recent reports indicate that MASLD is closely associated with obesity, cardiovascular disease, and type 2 diabetes (Cigrovski Berkovic et al., 2021). Moreover, complications of MASLD are often associated with increased intervention risks; for example, patients with MASLD and concomitant diabetes are at higher risk of cardiovascular events and hypoglycemia during exercise (Colberg et al., 2016). In addition, patient adherence is an important determinant of intervention effectiveness. Although there is a lack of studies on the long-term metabolic and cardiovascular effects of cold exposure, moderate-intensity, low-impact, and home-based exercise programs are more likely to be accepted and maintained by patients (Ricke et al., 2023). Therefore, in the early stage of intervention, excessive exercise intensity and cold exposure should not be required, and a gradual, stepwise approach should be followed. In summary, future clinical trials should enroll patients with early or moderate MASLD and adopt moderate-intensity aerobic exercise combined with resistance training. Randomized controlled trial designs should be implemented to improve the level of evidence, with interventions lasting 8–12 weeks, followed by long-term follow-up to continuously assess endpoints such as lipid metabolism and insulin sensitivity.

**Table 3:** Clinical feasibility and safety considerations for exercise, cold exposure, and combined intervention in MASLD.

Intervention	Risk profile	Cardiovascular impact	Clinical recommendation	References
Acute cold exposure (4–7 °C)	High sympathetic activation	↑ blood pressure, ↑ heart rate, vasoconstriction	Not recommended for high-risk patients	(Ikäheimo, 2018)
Moderate-intensity exercise (MICT)	Low risk	Cardioprotective adaptation	First-line therapy for MASLD	(Stine et al., 2023)
HIIT	Moderate risk	Transient increase in cardiac workload	Suitable only for stable patients	(Duncker and Bache, 2008; Stine et al., 2023)
Combined cold + exercise	Highest physiological stress	↑ myocardial oxygen demand, potential ischemia risk	Restricted to low-risk early MASLD patients	(Li et al., 2025b)

## 7 Limitations and future directions

Current evidence on combined exercise and cold exposure in MASLD remains limited. Most mechanistic evidence is derived from animal models, whereas human studies are mainly acute physiological studies conducted in non-MASLD populations and using surrogate molecular endpoints. Therefore, there is still a lack of well-designed randomized controlled trials investigating the efficacy, safety, optimal cold exposure temperature, exercise intensity, intervention duration, and long-term adherence of combined interventions in patients with MASLD. Future studies should enroll patients with early or moderate MASLD, stratify participants according to sex, menopausal status, cardiometabolic risk, and disease stage, and evaluate clinically relevant endpoints such as hepatic fat content, insulin sensitivity, lipid metabolism, inflammatory markers, fibrosis progression, cardiovascular safety, and long-term sustainability.

## 8 Conclusion

AMPK regulates multiple pathological processes in MASLD, including *de novo* lipogenesis (DNL), fatty acid β-oxidation, mitochondrial homeostasis, autophagy, oxidative stress, hepatic inflammation, and fibrosis progression. These effects are mediated mainly through AMPK/ACC/SREBP1c, AMPK/SIRT1/PGC-1α, and AMPK/mTOR/TFEB signaling axes. Exercise and cold exposure activate AMPK through distinct mechanisms, thereby exerting beneficial effects on MASLD. Acute exercise may induce transient contraction-related AMPK activation and improve insulin sensitivity, whereas long-term exercise may lead to more sustained adaptive changes in mitochondrial function, myokine release, insulin sensitivity, and hepatic lipid metabolism. In contrast, cold exposure primarily acts through sympathetic nervous system activation, BAT/iWAT thermogenesis, UCP1-mediated energy expenditure, and skeletal muscle cold adaptation, collectively enhancing substrate utilization and reducing hepatic lipid burden. The combination of exercise and cold exposure may produce more pronounced metabolic effects than either intervention alone by simultaneously enhancing contraction-induced signaling in skeletal muscle, thermogenic energy expenditure, inter-organ communication, and hepatic lipid oxidation. This combined strategy may improve MASLD through AMPK-related pathways involving PGC-1α/irisin, the FGF21–β-Klotho/FGFR1 axis, ACC, mTOR, and TFEB. However, current evidence regarding combined interventions for MASLD remains limited, particularly regarding optimal cold exposure temperature and exercise intensity. Future studies should include more human trials to determine optimal protocols, safety, and clinical efficacy, with a focus on the translational value of exercise combined with cold exposure in the prevention and treatment of MASLD, thereby providing a mechanistic basis for the development of non-pharmacological therapies for human MASLD.
